# Triglyceride is independently correlated with insulin resistance and islet beta cell function: a study in population with different glucose and lipid metabolism states

**DOI:** 10.1186/s12944-020-01303-w

**Published:** 2020-06-02

**Authors:** Minglei Ma, Haibin Liu, Jie Yu, Shuli He, Pingping Li, Chunxiao Ma, Huabing Zhang, Lingling Xu, Fan Ping, Wei Li, Qi Sun, Yuxiu Li

**Affiliations:** 1 Department of Endocrinology, Key Laboratory of Endocrinology, Ministry of Health, Peking Union Medical College Hospital, Beijing, 100730 China; 2 Department of Basic Physiology, The Health School affiliated with Capital Medical University, Beijing, China; 3 Department of Nutrition, Peking Union Medical College Hospital, Beijing, China; 4 State Key Laboratory of Bioactive Substance and Function of Natural Medicines, Institute of Materia Medica, Chinese Academy of Medical Sciences and Peking Union Medical College, Beijing, 100050 China; 5 Diabetes Research Center of the Chinese Academy of Medical Sciences, Beijing, 100050 China

**Keywords:** Glucose, Lipid, Diabetes, Triglyceride, Superoxide dismutase, Insulin resistance, Beta cell function, Oxidative stress

## Abstract

**Background:**

Previous studies on the effects of lipotoxicity and oxidative stress on islet beta cell function mainly focused on patients with diabetes, whereas studies on normal glucose tolerance (NGT) are few. The aim of this study was to explore the relationships among triglyceride (TG), high-density lipoprotein cholesterol (HDL-c), low-density lipoprotein cholesterol (LDL-c), oxidative stress indicators, insulin resistance, and beta cell function in populations with different glucose and lipid metabolism states.

**Methods:**

A total of 517 individuals were recruited from a rural community in Beijing, China. Glucose metabolism status was defined according to the results of a 75-g oral glucose tolerance test (OGTT). Dyslipidemia was defined as abnormal TG, HDL-c, or LDL-c levels. The population was divided into four groups: individuals with normal glucose and lipid levels (group A, *n* = 62); those with dyslipidemia alone (group B, *n* = 82); those with dysglycemia alone (group C, *n* = 121); and those with dysglycemia and dyslipidemia (group D, *n* = 247). Oxidative stress indicators, including superoxide dismutase (SOD), glutathione reductase (GR) and 8-hydroxydeoxyguanosine (8-OHdG), were measured. Homeostasis model assessment of insulin resistance (HOMA-IR) and glucose disposition index (DI_30_, DI_120_) were calculated to assess insulin resistance and islet beta cell function, respectively. Stratified multiple linear regression analysis was used to explore relationships between TG, HDL-c, LDL-c, oxidative stress indicators, and insulin resistance (natural log transformation of HOMA-IR, LnHOMA-IR) and beta cell function (natural log transformation of DI_30_, Ln DI_30_).

**Results:**

Compared with the control group, populations with dyslipidemia and/or dysglycemia showed significantly increased insulin resistance. Dyslipidemia aggravated insulin resistance and beta cell dysfunction in individuals with dysglycemia. Stratified regression analysis showed that TG positively correlated with LnHOMA-IR in individuals with normal glucose levels (beta = 0.321, 0.327, *P* = 0.011, 0.003 in groups A and B, respectively) and negatively correlated with LnDI_30_ in participants with dyslipidemia (beta = − 0.225, − 0.122, *P* = 0.035, 0.048 in groups B and D, respectively). Reduced serum SOD levels in individuals with dysglycemia plus dyslipidemia were observed, and a negative association between TG and SOD levels was found (r = − 0.461, *P* < 0.001).

**Conclusion:**

TG correlated with both insulin resistance and beta cell function in individuals with dyslipidemia alone. SOD negatively correlated with TG, indicating a close relationship between oxidative stress and glucose-lipid metabolism. Due to the adverse effect of hypertriglyceridemia on insulin sensitivity and islet beta cell function, more attention should be paid to the detection and management of hypertriglyceridemia.

## Background

As living conditions improve, lifestyle-related metabolic diseases, including diabetes (DM), obesity, dyslipidemia, and hyperuricemia, are becoming increasingly prevalent worldwide. Dyslipidemia is very common in patients with type 2 diabetes (T2DM), with a prevalence of 72–85% in this population [1]. According to previous studies, elevated triglyceride (TG) and decreased high-density lipoprotein cholesterol (HDL-c) are characteristic features of diabetic dyslipidemia [1, 2]. Patients with diabetes have an increased risk of cardiovascular disease (CVD), and low-density lipoprotein cholesterol (LDL-c) is an independent risk factor for CVD. Currently, lipid-lowering therapy for patients with diabetes primarily focuses on the reduction in LDL-c through the use of statins, while treatment aimed at lowering TG levels is relatively inadequate [3, 4].

Insulin resistance and impaired islet beta cell function are two major defects that contribute to the occurrence of T2DM. These defects may also contribute to the development of impaired fasting glucose (IFG) and impaired glucose tolerance (IGT), conditions that occur in the early stages of diabetes [5, 6]. As one component of metabolic syndrome, insulin resistance is closely related to obesity and dyslipidemia. Studies have shown that the TG/HDL-c ratio can be used to predict insulin resistance in Caucasian and Chinese Han populations [7–9], reflecting the close relationship among TG, HDL-c, and insulin resistance. Lipotoxicity can not only induce insulin resistance but also impair beta cell function. Both chronic hyperglycemia (glucotoxicity) and chronic hyperlipidemia (lipotoxicity) can impair islet beta cell function [10]. It has also been suggested that lipotoxicity damages islet beta cell function only under high-glucose conditions (glucolipotoxicity) [11–13]. Previous studies of lipotoxicity have primarily focused on diabetic mouse models or on patients with diabetes [14–16], whereas studies of individuals with normal glucose tolerance (NGT) are few [17].

Previous studies have shown that oxidative stress are involved in the pathogenesis of insulin resistance and islet beta cell dysfunction as well as in the development of diabetes [18–22]. Oxidative stress is the result of an imbalance between the formation and the enzymatic or nonenzymatic clearance of reactive oxygen species (ROS). Due to the low levels of antioxidant defense enzymes present in islets, beta cells are extremely susceptible to oxidative stress damage [23]. Very few studies have focused on the relationships among serum lipid levels, oxidative stress indicators, insulin resistance, and beta cell function in populations with different states of glucose and lipid metabolism, especially in individuals with hyperglycemia alone, dyslipidemia alone, or normal glucose and lipid metabolism.

It is hypothesized that TG, HDL-c, LDL-c and ROS might contribute differently to insulin resistance and islet beta cell dysfunction in people with different glucose and lipid levels. To address this issue, the relationship between serum lipid levels, oxidative stress indicators, insulin resistance, and islet beta cell function was explored in a previously healthy population residing in a rural community in Beijing, China.

## Methods

### Study population

**Fig. 1:**
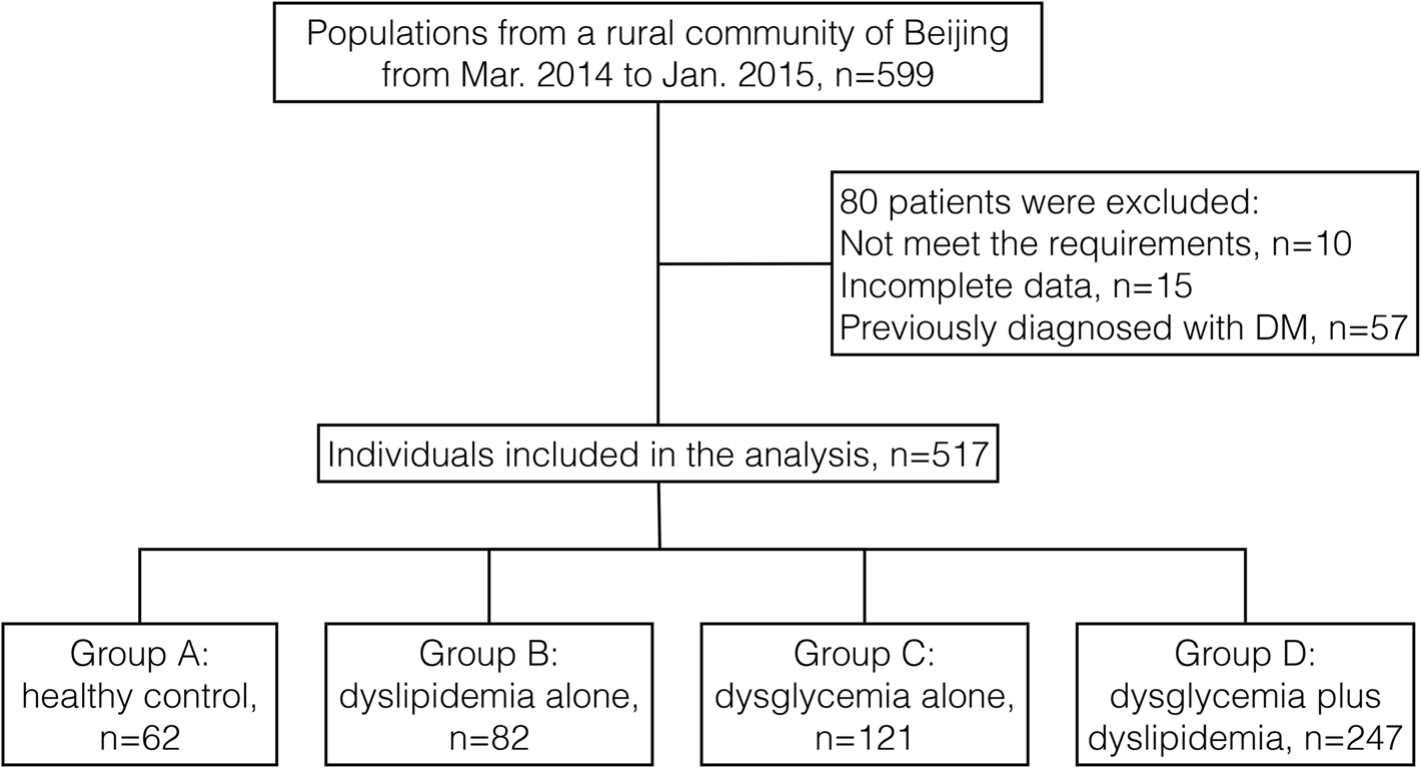
Flow chart of the study

This is a community-based cross-sectional study. The participants were recruited between March 2014 and January 2015 from a type 2 diabetes project in a suburb of Beijing, China. The study recruited participants from 599 participants who underwent a 75-g oral glucose tolerance test (OGTT). The exclusion criteria were as follows: 1) patients previously diagnosed with diabetes or who were taking hypoglycemic drugs (*n* = 57); 2) patients previously treated with steroid drugs or lipid-lowering drugs (*n* = 25, 22 of whom were also taking hypoglycemic drugs); and 3) patients with severe cardiovascular, hepatic, or kidney disease (*n* = 7). After excluding patients who did not meet the requirements (*n* = 67) and those for whom the data were incomplete (*n* = 15), 517 patients were included in the analysis (Fig. 1). The study protocol was approved by the Ethics Committee of Peking Union Medical College Hospital (Approval Number: ZS-1274), and all patients signed an informed consent form.

### Demographic data

Waist circumference (WC) was measured midway between the iliac crest and the costal margin. Height and weight were measured in patients without shoes wearing thin clothing and used to calculate the patient’s body mass index (BMI) according to the formula BMI = weight (kg)/height (m)^2^. According to the 2004 World Health Organization’s recommendation for Asian people, being overweight was defined as BMI ≥23 kg/m^2^ and obesity as BMI ≥27.5 kg/m^2^ [24]. Blood pressure at rest in the sitting position was measured twice with a standard mercury sphygmomanometer, and the average value was used.

### Biochemical test

Venous blood was collected through the elbow vein from participants who had fasted for at least 10 h (overnight fasting). The serum lipid profile, including total cholesterol (TC), TG, HDL-c, and LDL-c, was measured using a Beckman CX4 automatic biochemical analyzer (interassay coefficient of variation < 3%, testing performed with help from the laboratory staff at Peking Union Medical College Hospital). Glycosylated hemoglobin (HbA1c) was detected using the National Glycohemoglobin Standardization Program (NGSP) certification method. All patients underwent a 2-h OGTT in which they ingested 75 g of glucose powder. Venous blood was collected at 0, 30, 60, and 120 min and used to measure plasma glucose (glucose oxidase method), insulin (chemiluminescent enzyme immunoassay), and C peptide (C-P, chemiluminescent enzyme immunoassay) levels. The trapezoidal method was used to calculate the areas under the curves of glucose (AUC_Glu_), insulin (AUC_INS_) and C peptide (AUC_C-P_) from the OGTT.

### Oxidative stress indicators

Serum superoxide dismutase (SOD), glutathione reductase (GR), 8-hydroxydeoxyguanosine (8-OHdG) levels were determined using enzyme-linked immunosorbent assay (ELISA) kits (Cloud-Clone Corp, Houston, TX, USA); these measurements were conducted with help from Beijing North Institute of Biological Technology.

### Assessment of insulin resistance and beta cell function

Assessment of insulin resistance and insulin sensitivity was performed using the following models. (1) Homeostasis model assessment of insulin resistance (HOMA-IR): HOMA-IR = fasting blood glucose (mmol/L) × fasting insulin (μIU/mL)/22.5 [25]. HOMA-IR is determined from results on fasting indices and is mainly used to estimate hepatic insulin sensitivity. (2) Matsuda insulin sensitivity index (ISI_M_): ISI_M_ = 10^4^/[(fasting blood glucose × fasting insulin) × (average blood glucose × average insulin)]^0.5^ [26]. ISI_M_ can be used as a measure of whole-body insulin sensitivity. (3) Quantitative insulin sensitivity check index (QUICKI): QUICKI = 1/[log insulin (μIU/mL) + log glucose (mg/dL)] [27].

Evaluation of islet beta cell secretory function was performed as follows. (1) The insulinogenic index (IGI) was assessed for early-phase insulin secretion assessment (IGI_30_ = ΔINS_0–30_/ΔGlu_0–30_ [28]) and for late-phase insulin secretion (IGI_120_ = ΔINS_0–120_/ΔGlu_0–120_). (2) The disposition index (DI), a comprehensive evaluation of insulin secretion and insulin resistance, was measured as 1) beta cell function adjusted for insulin sensitivity: DI=HOMA-β/HOMA-IR [29]; 2) early-phase glucose DI: DI_30_ = ΔINS_0–30_/ΔGlu_0–30_ * ISI_M_; and 3) late-phase glucose DI: DI_120_ = ΔINS_0–120_/ΔGlu_0–120_ *ISI_M_ [30].

### Definition of glucose tolerance

According to the diagnostic criteria set forth by the 1999 World Health Organization (WHO), all patients were stratified for glucose tolerance; those with fasting plasma glucose (FPG) ≥7.0 mmol/L or 2-h postprandial plasma glucose (PPG) ≥11.0 mmol/L were defined as having DM, those with FPG ≥6.1 mmol/L but < 7.0 mmol/L or 2-h PPG ≥7.8 mmol/L but < 11.0 mmol/L were defined as having prediabetes (pre-DM), and those with FPG < 6.1 mmol/L and 2-h PPG < 7.8 mmol/L were defined as having NGT. Of the study participants, 118 (22.8%) were newly diagnosed with DM, 255 (49.3%) were diagnosed as having pre-DM, and 144 (27.9%) were diagnosed as having NGT.

### Definition of dyslipidemia

Dyslipidemia associated with T2DM is characterized by high TG and low HDL-c levels. In addition, T2DM is a high-risk factor for CVDs, and low LDL-c levels are closely related to the occurrence of CVDs. Therefore, the definition of dyslipidemia in this study is primarily based on abnormalities in TG and/or HDL-c or LDL-c levels. The definition of dyslipidemia used in this study was based on the definitions of abnormal TG and HDL-c levels in metabolic syndrome provided by the 2005 National Cholesterol Education Program (NCEP) and the 2006 International Diabetes Federation (IDF) and was indicated by a TG level ≥ 1.7 mmol/L, an HDL-c level < 1.03 mmol/L (male) or < 1.3 mmol/L (female), or an LDL-c level ≥ 3.37 mmol/L (based on the upper limit of the laboratory reference range). Among the participants, 192 (37.5%) had increased TG levels ≥1.7 mmol/L, 209 (40.8%) had decreased HDL-c levels < 1.0 mmol/L (male) or < 1.3 mmol/L (female), and 107 (20.9%) had increased LDL-c levels ≥3.37 mmol/L. Any single abnormality in TG, HDL-c, and LDL-c levels was defined as dyslipidemia. Among the participants, 329 (63.6%) had dyslipidemia, and 188 (36.3%) had normal serum lipids.

### Group of participants

Because the study did not include patients who had previously been diagnosed with DM, individuals with newly diagnosed DM and pre-DM were grouped together in the dysglycemia group. Further grouping was based on glucose tolerance and serum lipid levels. Therefore, the population was divided into the following four groups (due to partial data loss, four patients were not included in the group analysis): group A, healthy controls (normal glucose and lipid levels, *n* = 62); group B, individuals with dyslipidemia alone (dyslipidemia and NGT, *n* = 82); group C, individuals with dysglycemia alone (pre-DM or newly diagnosed DM and normal serum lipids, *n* = 121); and group D, individuals with both dysglycemia and dyslipidemia (pre-DM or newly diagnosed DM with dyslipidemia, *n* = 247) (Fig. 1).

### Statistical methods

Continuous data are expressed as the mean ± SD or as the median (Q1, Q3), and categorical variables are expressed as counts/percentiles (%). Normality was assessed for all continuous variables, and nonnormally distributed data were transformed using the natural log (Ln) value where relevant. Differences in normally distributed data among groups were assessed by one-way analysis of variance (ANOVA) with post hoc Bonferroni correction for multiple comparisons. Differences in nonnormally distributed data among groups were assessed using the nonparametric test, and *P* values comparing two groups were corrected by the Bonferroni method. Spearman correlation analysis was used to assess the association of different indicators with insulin resistance (HOMA-IR) and insulin secretion (DI_30_) in the overall population. In the multivariate linear regression analysis, the population was grouped according to states of glucose and lipid metabolism, and LnHOMA-IR and LnDI_30_ were used as dependent variables to evaluate the correlations among lipid profiles, oxidative stress indicators, insulin resistance, and beta cell function. Statistically significant variables (*P* < 0.2) from the univariate linear regression analyses were included in the multiple linear regression analysis. All statistical analyses were performed using IBM SPSS Statistics 23.0 (IBM Corp., Armonk, NY, USA). A *P* value < 0.05 was considered significant.

## Results

### Basic characteristics of the groups

The average age of the study population was 52.63 ± 11.08 years; individuals with dysglycemia (groups C and D) were on average older than those with NGT (groups A and B). Most individuals in groups B, C, and D were overweight or obese; the average BMI of the individuals in these groups was higher than that of group A. Among the individuals with normal serum lipid levels, those with dysglycemia (group C) showed a significant increase in LDL-c levels compared with those with NGT (group A). In participants with dyslipidemia, dysglycemic individuals (group D) had higher TG, TC, and LDL-c levels than participants with NGT (group B). The SOD level of group D was significantly reduced compared with that of group C (Table 1).

**Table 1:** Basic characteristics of the general population and of participants with different levels of glucose and lipid metabolism

		Group A	Group B	Group C	Group D	
	Total	NGT & NL	NGT & DL	IGT or DM & NL	IGT or DM & DL	
	*N* = 517	*N* = 62 (12.1%)	*N* = 82 (16.0%)	*N* = 121 (23.6%)	*N* = 247 (48.2%)	*P*
Female (%)	334 (65.2%)	43 (69.4%)	56 (68.3%)	69 (57.0%)	166 (67.2%)	0.185
Age (years)	52.63 ± 11.08	47.37 ± 12.54	48.35 ± 12.33	54.47 ± 10.52†‡	54.45 ± 9.70†‡	< 0.001
BMI (kg/m^2^)	26.07 ± 3.78	23.46 ± 3.06	25.57 ± 2.97†	25.48 ± 3.76†	27.18 ± 3.80†‡§	< 0.001
WC (cm)	87.22 ± 9.83	81.28 ± 9.51	85.11 ± 8.91	86.33 ± 9.52†	89.86 ± 9.50†‡§	< 0.001
Obesity	170 (33.1%)	6 (9.7%)	20 (24.4%)	35 (29.2%)	108 (43.9%)	< 0.001
Overweight or obesity	415 (80.7%)	32 (51.6%)	66 (80.5%)	95 (79.2%)	219 (89%)	< 0.001
SBP (mmHg)	127.71 ± 18.84	121.95 ± 16.55	123.44 ± 16.01	126.47 ± 20.15	131.17 ± 18.97†‡	< 0.001
DBP (mmHg)	76.15 ± 10.12	75.15 ± 10.28	76.85 ± 9.75	75.39 ± 9.29	76.54 ± 10.60	0.568
HbA1c (%)	5.6 (5.3, 6.0)	5.2 (5.0, 5.4)	5.3 (5.1, 5.6)	5.6 (5.3, 5.9) †‡	5.9 (5.5, 6.3) †‡§	< 0.001
FPG (mmol/L)	5.92 (5.46, 6.61)	5.27 (5.01, 5.39)	5.30 (5.10, 5.48)	6.07 (5.81,6.61) †‡	6.35 (5.86, 7.21) †‡	< 0.001
TG (mmol/L)	1.40 (0.98, 2.06)	0.91 (0.68, 1.20)	1.53 (0.99, 2.13) †	1.01 (0.8, 1.30) ‡	1.89 (1.39. 2.58) †‡§	< 0.001
HDL-c (mmol/L)	1.27 (1.08, 1.47)	1.47 (1.36, 1.61)	1.13 (1.01. 1.26) †	1.45 (1.32, 1.63) ‡	1.17 (1.03, 1.32) †§	< 0.001
TG/HDL-c	1.14 (0.71, 1.83)	0.64 (0.42, 0.83)	1.32 (0.83, 2.08) †	0.70 (0.52, 0.93) ‡	1.62 (1.18, 2.52) †‡§	< 0.001
TC (mmol/L)	5.49 ± 1.03	4.99 ± 0.61	5.35 ± 1.18	5.32 ± 0.81	5.73 ± 1.09†‡§	< 0.001
LDL-c (mmol/L)	2.84 ± 0.71	2.31 ± 0.46	2.85 ± 0.82†	2.61 ± 0.51†	3.08 ± 0.71†‡§	< 0.001
UA (μmol/L)	291.61 ± 83.38	266.09 ± 78.51	283.52 ± 71.90	275.89 ± 74.97	308.39 ± 88.77†	< 0.001
8-OHdG (pg/ml)	37.76 (19.82,58.66)	38.65 (18.72,58.49)	36.10 (17.86,62.76)	41.66 (25.02,55.35)	36.96 (19.66,59.80)	0.9
SOD level (U/ml)	58.95 ± 20.97	60.60 ± 15.73	59.95 ± 18.01	64.31 ± 17.27	55.71 ± 23.92§	0.003
GR (U/L)	7.19 ± 3.25	7.34 ± 3.33	6.59 ± 3.12	7.43 ± 3.19	7.25 ± 3.30	0.304

*BMI* Body mass index, *WC* Waist circumference, *SBP* Systolic blood pressure, *DBP* Diastolic blood pressure, *HbA1c* Glycosylated hemoglobin, *FPG* Fasting blood glucose, *TG* Triglyceride, *HDL-c* High-density lipoprotein cholesterol, *TC* total cholesterol, *LDL-c* Low-density lipoprotein cholesterol, *UA* Uric acid, *8-OHdG* 8-hydroxydeoxyguanosine, *SOD* Superoxide dismutase, *GR* Glutathione reductase.

†: *P* < 0.05 compared with group A

‡: *P* < 0.05 compared with group B

§: *P* < 0.05 compared with group C

### Comparison of glucose and insulin levels during the OGTT

**Fig. 2:**
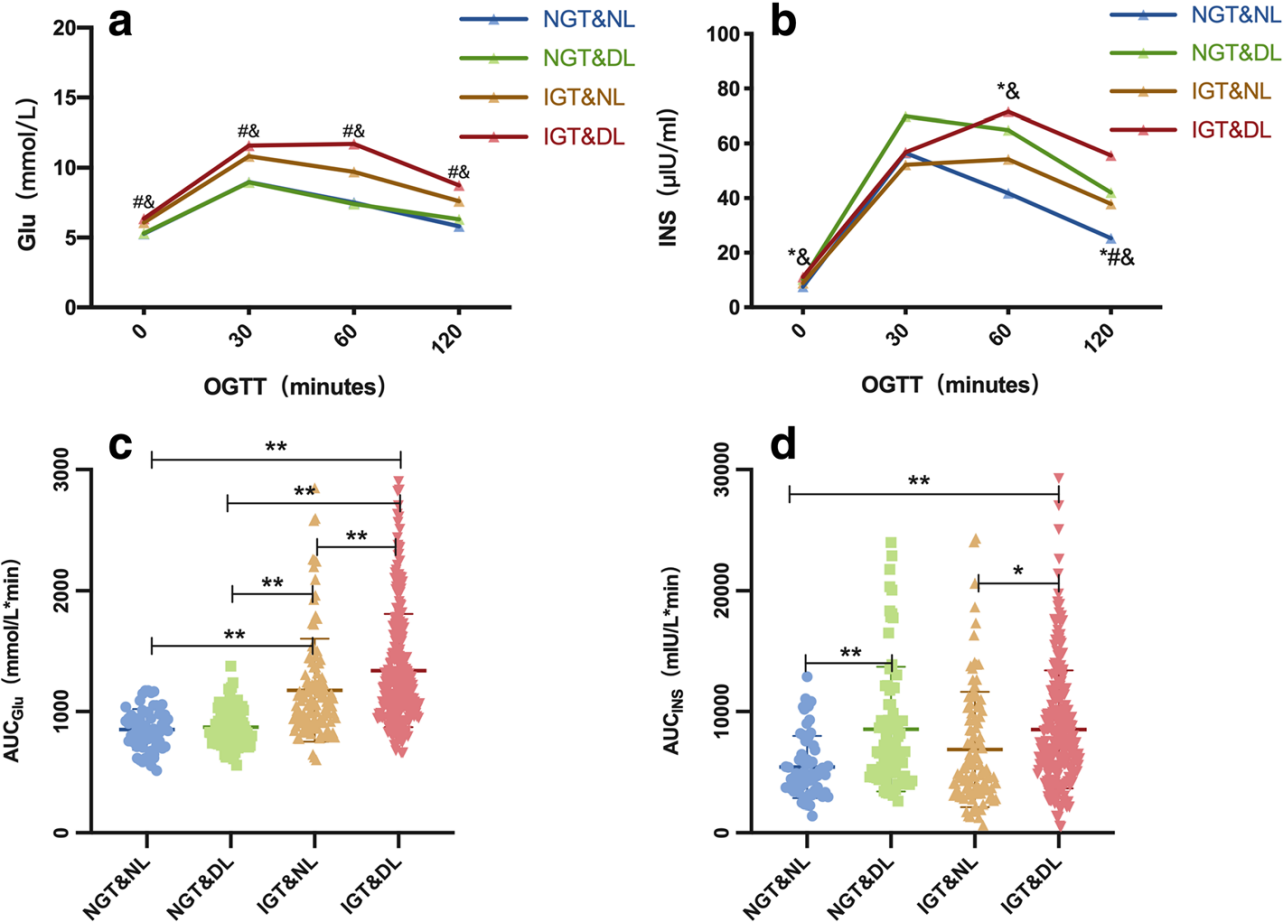
Comparison of blood glucose levels and insulin secretion in populations with different levels of glucose and lipid metabolism. **a**) Glucose levels and **b**) insulin levels at various time points during the OGTT; **c**) AUC of glucose and **d**) AUC of insulin during the OGTT. A/B: * *P* < 0.05 between group B and A, # *P* < 0.05 between group C and A, & *P* < 0.05 between group D and A. C/D: *P < 0.05, ** *P* < 0.001

Compared with the individuals in group A, the insulin levels of those in group B during fasting and at 30, 60, and 120 min of the OGTT were significantly increased, but the blood glucose levels of these two groups did not differ significantly at any of the time points. Group C showed a significant increase in blood glucose at various time points of the OGTT compared with group A, but there were no significant differences in the insulin levels of these two groups at 0, 30, or 60 min. The insulin level at 120 min was significantly higher than that in the control group. The AUC_Glu_ of the individuals in groups C and D was higher than that of those in groups A and B. The AUC_INS_ was higher in patients with dyslipidemia than in individuals with normal lipid levels both in patients with NGT and in those with pre-DM/DM (group B vs. group A and group D vs. group C) (Fig. 2, Suppl. Table 1).

### Comparison of insulin resistance and islet beta cell function among groups

**Fig. 3:**
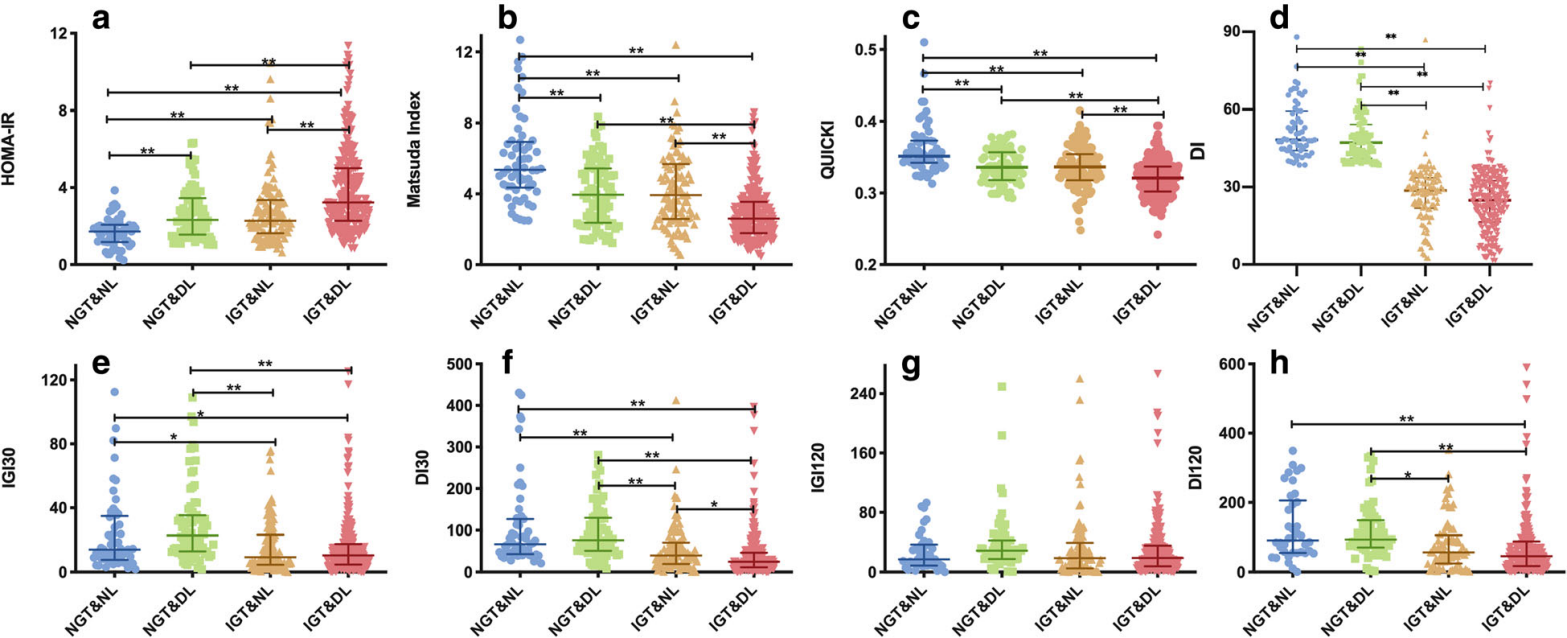
Comparison of insulin resistance and islet beta cell function in patients with different levels of glucose and lipid metabolism. **a**) HOMA-IR; **b**) ISI_M_; **c**) QUICKI; **d**) DI; **e**) IGI_30_; **f**) DI_30_; G) IGI_120_; H) DI_120_. HOMA-IR: homeostasis model assessment of insulin resistance; ISI_M_: Matsuda insulin sensitivity index; QUICKI: quantitative insulin sensitivity check index; DI: disposition index; IGI: insulinogenic index. * *P* < 0.05, ** *P* < 0.001

Compared with the normal control group (group A), groups B, C, and D all showed significantly higher HOMA-IR and significantly lower insulin sensitivity as indicated by QUICKI and ISI_M_. Compared with groups B and C, group D had more severe insulin resistance (HOMA-IR) and poorer insulin sensitivity (QUICKI and ISI_M_). However, there were no significant differences in HOMA-IR, QUICKI, or ISI_M_ between groups B and C (Fig. 3).

Group C showed significant decreases in IGI_30_ and DI_30_ compared with group B and group A and a significant decrease in DI_120_ compared with group B. Compared with group A, group B did not show significant differences in IGI_30_, DI_30_, IGI_120_, or DI_120_. Compared with groups A and B, group D showed significant decreases in IGI_30_, DI_30_, and DI_120_. Compared with group C, group D showed significant decreases in IGI_30_, DI30, and DI_120_ (Fig. 3).

### Differences in insulin sensitivity and DI among groups divided by quartiles with respect to the levels of lipid components

HOMA-IR was increased in participants who were in the 3rd and 4th quartiles for TG and LDL-c levels and decreased in those who were in the 4th quartile for HDL-c levels compared with those who were in the 1st quartile for these individual lipid components. Similarly, ISI decreased according to the quartiles in which the participants fell with respect to TG and LDL-c levels but increased in individuals who were in the 3rd and 4th quartiles for HDL-c (Suppl. Figure 1). DI declined in participants who were in the higher quartiles for TG and LDL-c but did not change significantly as a function of quartile in the case of HDL-c. DI_30_ was decreased in participants who were in the 3rd and 4th quartiles for TG; however, participants in different quartiles for HDL-c and LDL-c did not show significant differences in DI_30_ (Suppl. Figure 2).

### Relationship of serum lipid profiles and oxidative stress indicators to HOMA-IR and DI_30_

Spearman correlation analysis showed that TG, LDL-c, and UA positively correlated with HOMA-IR and negatively correlated with DI_30_, while HDL-c and SOD levels negatively correlated with HOMA-IR. In addition, HDL-c levels positively correlated with DI_30_, while 8-OHdG levels negatively correlated with DI_30_ (Suppl. Table 2). The stratified multiple linear regression analysis showed that TG positively correlated with insulin resistance (LnHOMA-IR) in the NGT groups (groups A and B) and negatively correlated with beta cell function (LnDI_30_) in the dyslipidemia groups (groups B and D). However, when adjustments were made for other confounding factors, HDL-c and LDL-c were no longer associated with insulin resistance or beta cell function (Tables 2 and 3). Then, partial correlation analysis was used to analyze the relationships between TG, HDL-c, LDL-c, and indicators of oxidative stress. The results indicated that TG negatively correlated with SOD level even after adjusting for age, gender, BMI, FPG, and UA (r = − 0.461, *P* < 0.001). In addition, GR independently positively correlated with LnDI_30_, while 8-OHdG was independently negatively correlated with LnDI_30_ in the individuals in group A (Table 3).

**Table 2:** Regression analysis using LnHOMA-IR as a dependent variable in groups with different glucose and lipid levels

	Simple linear regression			Multiple linear regression				
Group A	B	Beta	*P*	B	Beta	*P*	VIF	
Age	−0.019	− 0.443	< 0.001	−0.018	− 0.415	< 0.001	1.361	
Gender	0.364	0.31	0.014	0.145	0.124	0.198	1.373	
BMI (kg/m^2^)	0.065	0.362	0.004	0.045	0.254	0.005	1.161	
FPG (mmol/L)	0.462	0.528	< 0.001	0.398	0.454	< 0.001	1.056	
TG (mmol/L)	0.546	0.321	0.011	0.412	0.242	0.007	1.136	
Adjusted R2								0.599
Group B	B	Beta	*P*	B	Beta	*P*	VIF	
Age	−0.008	−0.201	0.076	−0.01	− 0.241	0.018	1.125	
BMI (kg/m^2^)	0.066	0.407	< 0.001	0.055	0.331	0.004	1.408	
WC (cm)	0.016	0.3	0.007	0.006	0.061	0.596	1.499	
FPG (mmol/L)	0.354	0.19	0.09	0.249	0.132	0.182	1.081	
TG (mmol/L)	0.161	0.327	0.003	0.053	0.288	0.006	1.181	
LDL-C (mmol/L)	0.114	0.194	0.083	0.062	0.08	0.451	1.266	
GR (U/L)	0.031	0.2	0.073	0.016	0.096	0.343	1.13	
Adjusted R2								0.307
Group C	B	Beta	*P*	B	Beta	*P*	VIF	
Gender	0.237	0.192	0.037	0.333	0.269	0.003	1.323	
BMI (kg/m^2^)	0.075	0.462	< 0.001	0.074	0.457	< 0.001	2.184	
WC (cm)	0.012	0.18	0.053	−0.007	−0.107	0.344	2.098	
FPG (mmol/L)	0.089	0.032	0.006	0.068	0.192	0.022	1.122	
TG (mmol/L)	0.494	0.244	0.008	0.032	0.016	0.86	1.283	
HDL-c (mmol/L)	−0.627	−0.225	0.015	−0.526	− 0.185	0.062	1.585	
Adjusted R2								0.302
Group D	B	Beta	*P*	B	Beta	*P*	VIF	
BMI (kg/m^2^)	0.059	0.396	< 0.001	0.048	0.317	< 0.001	1.394	
WC (cm)	0.019	0.309	< 0.001	0.005	0.081	0.204	1.406	
FPG (mmol/L)	0.105	0.39	< 0.001	0.114	0.394	< 0.001	1.092	
TG (mmol/L)	0.066	0.235	< 0.001	0.013	0.046	0.516	1.744	
SOD (U/mL)	−0.004	−0.184	0.005	−0.001	−0.042	0.545	1.704	
Adjusted R2								0.33

*BMI* Body mass index, *WC* Waist circumference, *FPG* Fasting blood glucose, *TG* Triglyceride, *HDL-c* High-density lipoprotein, *LDL-c* Low-density lipoprotein, *SOD* Superoxide dismutase, *GR* Glutathione reductase

**Table 3:** Regression analysis using LnDI_30_ as a dependent variable in groups with different levels of glucose and lipid metabolism

	Simple linear regression			Multiple linear regression				
Group A	B	Beta	*P*	B	Beta	*P*	VIF	
Gender	0.782	0.387	0.002	0.623	0.307	0.022	1.322	
Age	−0.023	−0.295	0.024	−0.01	−0.13	0.322	1.331	
WC (cm)	−0.023	−0.225	0.089	−0.015	− 0.144	0.234	1.133	
LDL-C (mmol/L)	−0.575	−0.275	0.035	−0.178	− 0.085	0.506	1.261	
GR (U/L)	0.094	0.312	0.016	0.079	0.262	0.027	1.045	
8-OHdG (pg/mL)	−0.005	−0.196	0.138	−0.007	−0.287	0.018	1.097	
Adjusted R2*								0.277
Group B	B	Beta	*P*	B	Beta	*P*	VIF	
Gender	0.724	0.376	0.001	0.579	0.295	0.011	1.204	
Age	−0.014	−0.203	0.085	−0.014	−0.202	0.058	1.041	
TG (mmol/L)	−0.195	−0.22	0.057	−0.214	− 0.228	0.034	1.054	
HDL-c (mmol/L)	1.102	0.266	0.021	0.916	0.218	0.054	1.068	
UA (μmol/L)	−0.003	−0.236	0.042	−0.001	−0.098	0.39	1.209	
Adjusted R2								0.243
Group C	B	Beta	*P*	B	Beta	*P*	VIF	
Age	−0.026	−0.236	0.017	−0.021	−0.188	0.013	1.019	
TG (mmol/L)	−0.536	−0.721	< 0.001	− 0.114	− 0.031	0.685	1.082	
FPG (mmol/L)	−0.697	−0.179	0.071	−0.555	− 0.645	< 0.001	1.084	
GR (U/L)	0.057	0.169	0.099	0.032	0.093	0.224	1.053	
Adjusted R2								0.488
Group D	B	Beta	*P*	B	Beta	*P*	VIF	
WC (cm)	−0.025	−0.184	0.006	−0.009	−0.068	0.142	1.088	
FPG (mmol/L)	−0.43	−0.731	< 0.001	− 0.439	− 0.693	< 0.001	1.153	
TG (mmol/L)	−0.172	−0.285	< 0.001	− 0.073	− 0.122	0.048	1.916	
HDL-C (mmol/L)	−0.65	−0.165	0.012	−0.157	− 0.04	0.391	1.107	
UA (μmol/L)	0.002	0.113	0.088	0.001	0.07	0.138	1.114	
SOD (U/mL)	0.012	0.23	< 0.001	−0.001	−0.012	0.842	1.75	
Adjusted R2								0.562

*BMI* Body mass index, *WC* Waist circumference, *FPG* Fasting blood glucose, *TG* Triglyceride, *HDL-c* High-density lipoprotein, *LDL-c* Low-density lipoprotein, *UA* Uric acid, *SOD* Superoxide dismutase, *GR* Glutathione reductase, *8-OHdG* 8-hydroxydeoxyguanosine

## Discussion

In this study, cross-group analysis showed increased insulin resistance in patients with dyslipidemia and/or dysglycemia. Although insulin resistance was observed in people with dyslipidemia alone, islet beta cell function was sufficiently preserved to allow these individuals to maintain normal glucose homeostasis. Multiple linear regression analysis showed that TG positively correlated with insulin resistance in participants with NGT (groups A and B) and negatively correlated with beta cell function in individuals with dyslipidemia (groups B and D) independent of other confounding factors. These results suggest that in populations with different blood glucose and lipid levels, the factors that contribute to insulin resistance and beta cell dysfunction are different and that the mechanisms involved might also be different.

In this study, 72.1% of the presumed “healthy” population in the rural community actually displayed abnormal glucose tolerance (22.8% had DM, and 49.3% had pre-DM), and 64.3% of the individuals tested had dyslipidemia. The prevalence of DM was higher than that reported by a previous study, which found that the prevalence of DM in the Chinese adult population was 11.6%, while the prevalence of pre-DM (50.1%) in that study was similar [31]. Additionally, the prevalence of dyslipidemia was substantially higher than those in previous studies that reported prevalence rates of 32.2 to 43.2% in Chinese rural adults [32, 33]. However, another study showed that the prevalence of dyslipidemia is 60.7 and 66.4% in rural and urban northeast Chinese adults aged over 40 years, respectively [34]. One important reason why that study and the present study showed a substantially higher prevalence of dyslipidemia might be due to the relatively older age of the participants. Hence, the prevalence of DM/pre-DM and dyslipidemia of the elderly in the Beijing rural community is high, while the rate of awareness is low. Therefore, more attention is needed to detect and manage DM/pre-DM and dyslipidemia in this population.

Patients with dyslipidemia exhibited severe insulin resistance compared with those without dyslipidemia, with or without dysglycemia. When both abnormalities were present, the degree of insulin resistance was further aggravated. These results are partially in accordance with those reported in a previous study in which elevated HOMA-IR was found in people with dyslipidemia alone [35]. Multiple linear regression analysis after population stratification showed that TG positively correlated with insulin resistance only in individuals with NGT (groups A and B). That is, in the absence of dysglycemia, an elevated TG level might be an important independent risk factor for insulin resistance. A previous study showed that both fenofibrate alone and the combination of omega-3 fatty acids and fenofibrate not only decreased triglycerides but also improved insulin sensitivity in patients with hypertriglyceridemia [36], thus showing a potential benefit of improving insulin resistance by combating hypertriglyceridemia. Therefore, the monitoring and control of hypertriglyceridemia in patients with dyslipidemia should be strengthened to improve insulin resistance.

Multiple linear regression analysis showed that TG was independently and negatively correlated with LnDI_30_ in patients with dyslipidemia (groups B and D), suggesting that an increased TG level is an independent risk factor for beta cell dysfunction in patients with dyslipidemia. Compared with the patients in group B, those in group D had higher TG levels, more severe insulin resistance, and significantly impaired early- and late-phase islet beta cell function. That is, as TG levels increase, insulin resistance and insulin secretion deficiency might worsen, and dysglycemia might occur. A previous T2DM cohort study found that log (TG/HDL-c) predicted the rate of decline in islet beta cell function [37]. In another study, Andrea Natali et al. reported that high TG and low HDL-c levels might be risk factors contributing to reduced insulin secretion in a nondiabetic cohort [17], a finding that is partially consistent with the results of the present study. However, in this study, HDL-c levels did not correlate with insulin resistance or beta cell function after adjustment for confounding factors. These discrepant results might be the consequences of differences in grouping and in adjustments for confounding factors in the multiple regression analysis.

As shown above, TG levels positively correlated with insulin resistance in individuals with NGT and negatively correlated with beta cell function in patients with dyslipidemia. That is, in patients with NGT associated with dyslipidemia, elevated TG not only aggravated insulin resistance but also accelerated islet beta cell dysfunction. Therefore, for patients with dyslipidemia, even if the blood glucose is normal, the management of hypertriglyceridemia should be strengthened. In addition to lifestyle modifications and the use of drugs such as fibrates, niacin, and n-3 fatty acids to combat hypertriglyceridemia, as recommended by Endocrine Society Clinical Practice Guidelines [38], certain nutraceuticals, such as fish oil, grape seed, mulberry, soy milk, and green, oolong, black, and pu-erh teas, might also be effective in reducing TG levels [39]. Furthermore, two randomized controlled trial studies conducted in India showed that saroglitazar, a dual peroxisome proliferator-activated receptor (PPAR) α/훾 agonist, lowers postprandial TG levels [40] and improves insulin sensitivity assessed using hyperinsulinemic-euglycemic clamp in patients with T2DM and hypertriglyceridemia [41]. These findings demonstrate the potential benefit and significant value of treatment of hypertriglyceridemia in improving insulin resistance.

As the primary antioxidant enzyme in the body, SOD removes ROS and reduces oxidative damage to cells and tissues. In this study, patients with dysglycemia plus dyslipidemia (group D) presented with significantly decreased SOD levels. In addition, SOD levels negatively correlated with insulin resistance, although the association disappeared after adjustment for confounding factors, including TG. However, TG was negatively correlated with SOD levels after adjustment for confounding factors, indicating that SOD might be involved in the connection between TG levels and insulin resistance. These results suggest that dyslipidemia might decrease SOD activity in patients with dysglycemia, aggravating oxidative stress damage and thereby impairing beta cell function. Another possibility is that the decreased SOD activity might induce hypertriglyceridemia, thereby aggravating insulin resistance and beta cell dysfunction. Some studies have demonstrated decreased SOD levels in patients with diabetes [42, 43], while other studies have reported increased or unchanged SOD levels in diabetic patients [44, 45]. A cross-sectional study showed that serum SOD level negatively correlates with BMI, TG, BG, and carotid artery intima-media thickness [46], in accordance with the present study. Perriotte-Olson et al. found that mice fed a high-fat diet and treated with nanoformulated SOD (NanoSOD) showed significant decreases in plasma and liver TG levels [47], and improved glucose and lipid metabolism [48]. The evidence above indicates that SOD plays an important role in glucose and lipid metabolism, and the mechanism of its effect requires further study.

Reduced glutathione (GSH) and GR are also important components of the antioxidant system in the body. 8-OHdG is a product of DNA damage caused by ROS [49] and can be used as a sensitive marker of DNA oxidative damage [50, 51]. Previous studies have reported that serum GSH levels and GR activity are decreased in patients with T2DM [45], and that the levels of 8-OHdG in blood and urine are significantly higher in diabetic or prediabetic populations [52–54]. In the present study, stratified regression analysis revealed that GR activity positively correlated with islet beta cell function and that 8-OHdG negatively correlated with beta cell function in individuals with normal blood glucose and lipid levels. These results suggest that GR plays an important role in maintaining normal islet beta cell function, whereas oxidative stress might impair beta cell function in the early stage, when hyperglycemia has not yet occurred. Therefore, paying attention to the role of oxidative stress prior to the occurrence of DM and dyslipidemia is promising and helpful. Hence, the relationship between oxidative stress and glucose-lipid metabolism is complex, and further investigation is needed to confirm the causality.

### Strengths and limitation

The strengths of this study are as follows. The population was grouped according to the patients’ levels of glucose and lipid metabolism and was also stratified in the regression analysis to avoid unrealistic analytical results based on an unstratified population. In addition to the results of the glucose tolerance test and the lipid profiles, oxidative stress indicators were included in the analysis, allowing us to rule out the impact of relevant confounding factors on the results. This study also has disadvantages and limitations. It is a cross-sectional study, and the conclusions obtained cannot be used to infer causality and must be further confirmed in follow-up cohort studies. In addition, no differences in GR activity or 8-OHdG levels among the four groups were found, possibly due to the relatively small sample size after grouping.

## Conclusions

TG levels positively correlated with insulin resistance in individuals with NGT and negatively correlated with beta cell function in people with dyslipidemia. Therefore, it is necessary to strengthen the control of hypertriglyceridemia in individuals with dyslipidemia, even when the glucose tolerance test is normal. In addition, oxidative stress might affect islet cell function at an early stage prior to the occurrence of abnormal glucose and lipid metabolism, which requires further investigation to gain a better understanding of the mechanisms that lead to DM as well as to arrest DM at an early stage.

## Supplementary information

**Additional file 1: Table S1.** Comparisons of blood glucose levels and insulin secretion at various time points during the OGTT for the general population and for populations with different levels of glucose and lipid metabolism. **Table S2.** Spearman correlation analysis of blood glucose, serum lipid levels, and oxidative stress indicators with HOMA-IR and DI_30_ in the general population. **Figure S1.** Comparison of HOMA-IR and ISI_M_ in patients according to quartiles of lipid profiles. * *P* < 0.05, ** *P* < 0.001.

## Data Availability

The datasets used and/or analyzed in the current study are available from the corresponding author upon reasonable request.

## Abbreviations

NGT: Normal glucose tolerance
IFG: Impaired fasting glucose
IGT: Impaired glucose tolerance
Pre-DM: Prediabetes mellitus
T2DM: Type 2 diabetes mellitus
CVD: Cardiovascular disease
WC: Waist circumference
SBP: Systolic blood pressure
DBP: Diastolic blood pressure
BMI: Body mass index
OGTT: Oral glucose tolerance test
FPG: Fasting plasma glucose
HbA1c: Glycosylated hemoglobin
TG: Triglyceride
TC: Total cholesterol
HDL-c: High-density lipoprotein cholesterol
LDL-c: Low-density lipoprotein cholesterol
UA: Uric acid
ROS: Reactive oxygen species
SOD: Superoxide dismutase
GR: Glutathione reductase
GSH: Reduced glutathione
8-OHdG: 8-hydroxydeoxyguanosine
HOMA-IR: Homeostasis model assessment of insulin resistance
ISI_M_: Matsuda insulin sensitivity index
QUICKI: Quantitative insulin sensitivity check index
IGI: Insulinogenic index
DI: Disposition index
Ln: Natural log transformation
