# Supplementary material for: Triglyceride is independently correlated with insulin resistance and islet beta cell function: a study in population with different glucose and lipid metabolism states
Source: Lipids Health Dis. 2020 Jun 2;19:121. doi: 10.1186/s12944-020-01303-w (PMC7268278; doi:10.1186/s12944-020-01303-w)
Supplement: Supplementary file 1 — Additional file 1: Table S1. Comparisons of blood glucose levels and insulin secretion at various time points during the OGTT for the general population and for populations with different levels of glucose and lipid metabolism. Table S2. Spearman correlation analysis of blood glucose, serum lipid levels, and oxidative stress indicators with HOMA-IR and DI30 in the general population. Figure S1. Comparison of HOMA-IR and ISIM in patients according to quartiles of lipid profiles. * P < 0.05, ** P < 0.001. [file 12944_2020_1303_MOESM1_ESM.docx]

Suppl. Table 1: Comparisons of blood glucose levels and insulin secretion at various time points during the OGTT for the general population and for populations with different levels of glucose and lipid metabolism

|  | Total | Group A, NGT & NL | Group B, NGT & DL | Group C, IGT & NL | Group D, IGT & DL | *P* |
| --- | --- | --- | --- | --- | --- | --- |
|  | n=517 | n=62 | n=82 | n=121 | n=247 |  |
| FPG (mmol/L) | 5.92 (5.46, 6.61) | 5.27 (5.01, 5.39) | 5.30 (5.10, 5.48) | 6.07 (5.81, 6.61) †‡ | 6.35 (5.86, 7.21) †‡ | <0.001 |
| Glu 30 (mmol/L) | 10.50 (8.94, 12.65) | 8.98 (7.23, 10.20) | 8.96 (7.21, 10.06) | 10.83 (9.22, 12.64) †‡ | 11.59 (9.90, 13.95) †‡§ | <0.001 |
| Glu 60 (mmol/L) | 9.75 (7.36, 13.31) | 7.50, (5.80, 9.17) | 7.41 (5.97, 9.38) | 9.71 (7.56, 13.67) †‡ | 11.71 (8.82, 15.65) †‡§ | <0.001 |
| Glu 120 (mmol/L) | 7.31 (5.86, 9.82) | 5.81 (4.66, 6.74) | 6.30 (5.01, 7.03) | 7.60 (5.84, 9.41) †‡ | 8.74 (6.82, 12.01) †‡§ | <0.001 |
| AUC_Glu_ (mmol/L*min) | 1,051.43 (873.79, 1,315.16) | 834.98 (728.29, 972.98) | 847.95 (751.54, 989.18) | 1,064.93 (914.70, 1,274.44) †‡ | 1,208.85 (1,008.94, 1,585.65) †‡§ | <0.001 |
| INS 0 (mIU/L) | 9.65 (6.75, 14.06) | 7.56 (5.32, 8.84) | 10.24 (6.75, 15.19) † | 8.89 (5.36, 12.73) | 11.12 (7.95, 16.31) †§ | <0.001 |
| INS 30 (mIU/L) | 56.16 (38.41, 96.08) | 56.50 (40.43, 79.35) | 69.89 (47.35, 149.07) | 52.24 (31.16, 82.38) ‡ | 56.78 (39.47, 93.16) ‡ | 0.003 |
| INS 60 (mIU/L) | 61.30 (39.78, 108.60) | 41.79 (32.58, 60.98) | 64.87 (43.13, 106.09) † | 54.20 (33.90, 94.02) | 71.57 (46.99, 122.02) †§ | <0.001 |
| INS 120 (mIU/L) | 43.24 (23.71, 68.61) | 25.25 (14.11, 34.74) | 42.05 (22.00, 58.90) † | 37.84 (16.02, 57.03) † | 55.59 (34.45, 83.11) †‡§ | <0.001 |
| AUC_INS_ (mIU/L*min) | 6,427.5 (4,276.35, 9,890.85) | 4,751.85 (3,492.26, 6,732.83) | 6,725.63 (4,833.98, 1,0584.04) † | 5,209.65 (3,462.00, 9,456.00) ‡ | 7,454.70 (5,303.05, 1,134.43) †§ | <0.001 |
| C-P 0 (ng/mL) | 1.32 (1.00, 1.77) | 0.98 (0.82, 1.21) | 1.28 (1.01, 1.67) † | 1.19 (0.92, 1.48) † | 1.51 (1.19, 2.14) †‡§ | <0.001 |
| C-P 30 (ng/mL) | 4.37 (3.30, 6.04) | 4.26 (3.61, 5.96) | 5.30 (3.96, 8.05) | 4.10 (2.82, 5.75) ‡ | 4.34 (3.29, 5.69) ‡ | <0.001 |
| C-P 60 (ng/mL) | 5.75 (4.21, 7.89) | 4.86 (3.51, 6.17) | 6.16 (4.30, 8.12) † | 5.29 (3.74, 7.42) | 6.39 (4.53, 8.22) †§ | <0.001 |
| C-P 120 (ng/mL) | 5.34 (3.68, 7.34) | 3.83 (3.02, 4.91) | 4.90 (3.38, 6.52) † | 4.64 (3.11, 7.12) † | 6.28 (4.32, 8.22) †‡§ | <0.001 |
| AUC_C-P_  (ng/mL*min) | 584.66 (451.84, 761.41) | 488.39 (404.51, 569.10) | 630.60 (490.41, 779.24) † | 531.15 (390.45, 754.05) | 632.10 (503.40, 810.15) †§ | <0.001 |

Note: FPG: fasting plasma glucose; Glu: glucose; INS: insulin; C-P: C-peptide; AUC: area under the curve. †: *P*<0.05 compared with group A, ‡: *P*<0.05 compared with group B, §: *P*<0.05 compared with group C.

Suppl. Table 2: Spearman correlation analysis of blood glucose, serum lipid levels, and oxidative stress indicators with HOMA-IR and DI_30_ in the general population.

|  | HOMA-IR |  | DI_30_ |  |
| --- | --- | --- | --- | --- |
|  | rho | *P* | rho | *P* |
| age | -0.037 | 0.406 | -0.21 | <0.001 |
| BMI | 0.515 | <0.001 | -0.149 | 0.001 |
| WC | 0.331 | <0.001 | -0.186 | <0.001 |
| FPG | 0.472 | <0.001 | -0.646 | <0.001 |
| HbA1c | 0.342 | <0.001 | -0.619 | <0.001 |
| TG | 0.416 | <0.001 | -0.288 | <0.001 |
| HDL-c | -0.246 | <0.001 | 0.127 | 0.006 |
| LDL-c | 0.194 | <0.001 | -0.156 | 0.001 |
| UA | 0.165 | <0.001 | -0.104 | 0.025 |
| SOD | -0.156 | <0.001 | 0.083 | 0.074 |
| GR | 0.046 | 0.311 | 0.038 | 0.418 |
| 8-OHdG | -0.006 | 0.892 | -0.101 | 0.029 |

Note: BMI: body mass index; WC: waist circumference; FPG: fasting blood glucose; HbA1c: glycosylated hemoglobin; TG: triglyceride; HDL-c: high-density lipoprotein; LDL-c: low-density lipoprotein; UA: uric acid; SOD: superoxide dismutase; GR: glutathione reductase; 8-OHdG: 8-hydroxydeoxyguanosine.


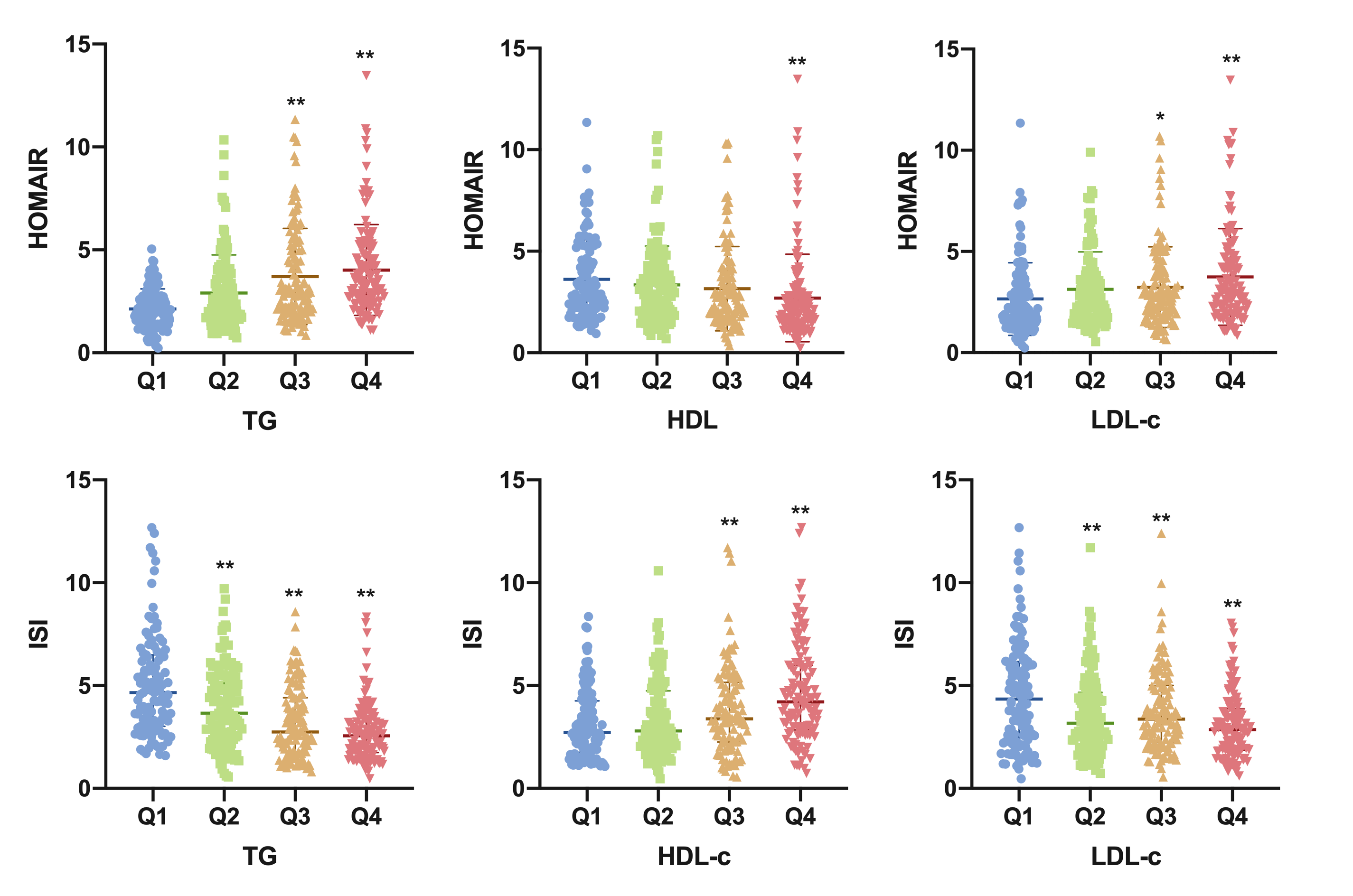


Suppl. Figure 1 Comparison of HOMA-IR and ISI_M_ in patients according to quartiles of lipid profiles. * *P*<0.05, ** *P*<0.001.


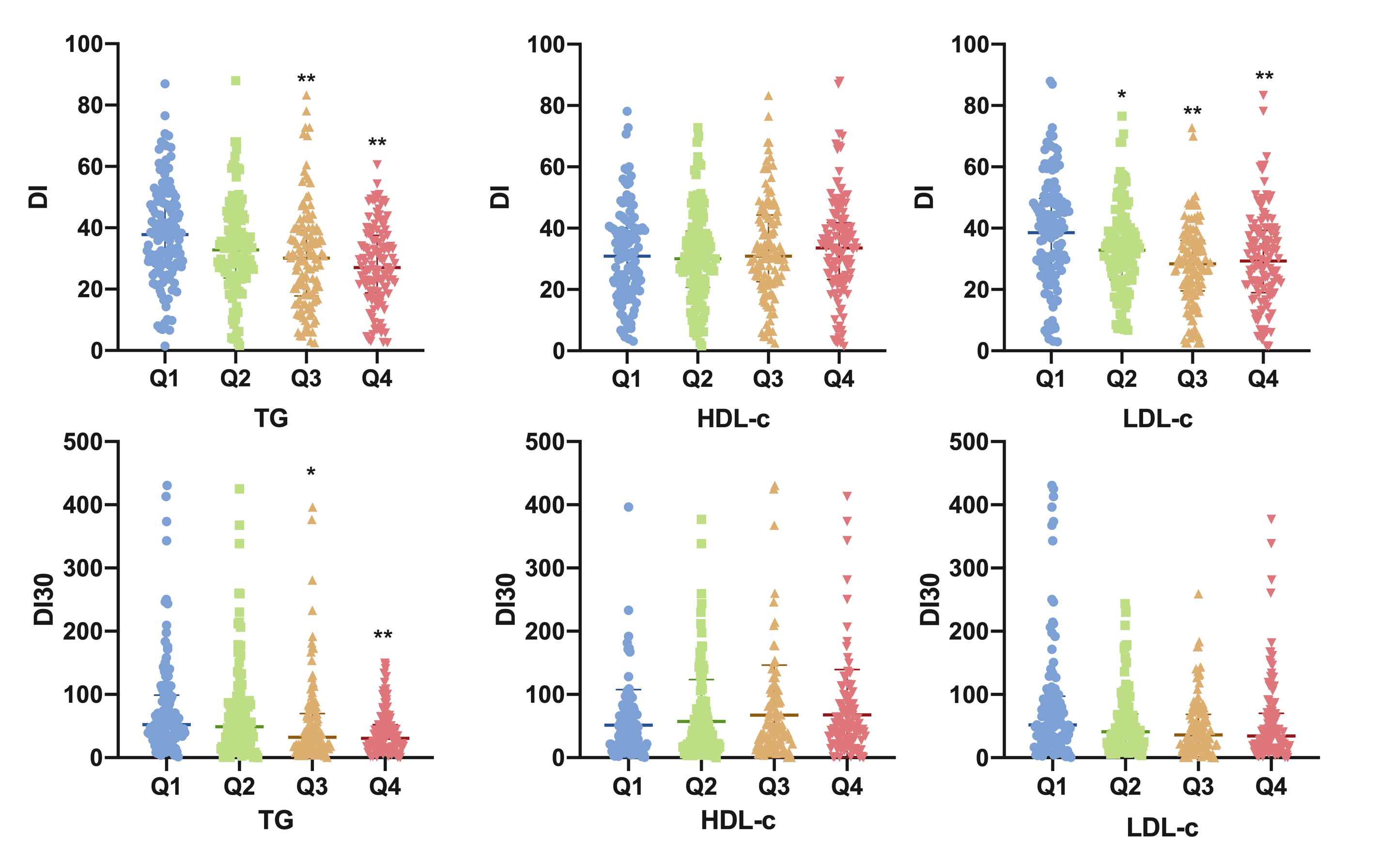


Suppl. Figure 2 Comparisons of DI and DI_30_ in patients according to quartiles of lipid profiles. * *P*<0.05, ** *P*<0.001.
